# Herpes simplex keratitis revisited

**DOI:** 10.5935/0004-2749.20210082

**Published:** 2021

**Authors:** Sidney Júlio Faria-e-Sousa, Rosalia Antunes-Foschini

**Affiliations:** 1 Department of Ophthalmology, Otorhinolaryngology, and Head and Neck Surgery, Medical School of Ribeirão Preto, University of São Paulo, Ribeirão Preto, São Paulo, Brazil Hospital das Clínicas, Medical School of Ribeirão Preto, University of São Paulo, Ribeirão Preto, São Paulo, Brazil; 2 Hospital das Clínicas, Medical School of Ribeirão Preto, University of São Paulo, Ribeirão Preto, São Paulo, Brazil

**Keywords:** Keratitis, herpetic/complications, Herpes simplex/ microbiology, Anti-bacterial agents/therapeutic use, Ceratite herpética/complicações, Herpes simples/ microbiologia, Antibacterianos/uso terapêutico

## Abstract

The degree to which viral infection and the host’s immune reaction to viral particles participate in the inflammatory process across various forms of herpetic keratitis has remained controversial. This fact has created conflicts regarding the classification of and therapeutic planning for such morbidities. This review aims to stimulate reflection on the classifications’ adequacy, nomenclatures, and therapeutic approaches related to these entities.

## Abstract (pt)

O grau de participação da infecção viral e da reação imunológica do hospedeiro às partículas virais no processo infamatório das diferentes formas de ceratites herpéticas ainda é objeto de controvérsia. Esse fato gera conflitos de classificação e planejamento terapêutico relativos a essas morbidades. Esta revisão visa estimular a reflexão sobre a adequabilidade das classificações, nomenclaturas e abordagens terapêuticas dessas entidades.

## INTRODUCTION

Ocular herpes, the most common cause of corneal blindness in developed countries, significantly impact life quality^(1,2)^. Herpes simplex virus type 1 infects about 50% of the United States population aged 30 years and 100% of those aged 60^(3)^. The Herpesviridae family comprises DNA viruses whose only natural host is the human species, including Cytomegalovirus; Epstein-Barr; Herpesvirus 6, 7, and 8; Varicella-Zoster; and Herpes simplex viruses^(4)^. However, this revision only concerns corneal diseases associated with the herpes simplex virus (HSV).

The most common clinical manifestations of HSV are herpes labialis, gingivostomatitis, and genital infections. Immunologically and virologically, two types of HSV are distinguished, namely, type 1 and type 2. HSV-1 commonly manifests in the oral cavity, lips, and ocular surface, with contamination occurring through contact with active lip vesicular lesions or asymptomatic patients’ saliva. HSV-2, which usually affects the genitals, is generally transmitted through sexual activity. Although HSV-2 can occasionally infect adults’ eyes through contaminated genital secretions, eye infections occur more often among neonates during passage through the birth canal.

The primary infection is usually unnoticed. The virus colonizes the trigeminal (Type 1) or spinal (Type 2) ganglia through viremia and becomes latent and therapeutically invulnerable at these places. The typical sign of primary HSV-1 infection is herpes labialis. In cases with eye involvement, the following clinical characteristics can be present: (1) follicular conjunctivitis, usually monocular, which persists for two weeks; (2) ipsilateral preauricular adenopathy; (3) small, transient corneal dendritic lesions of late-onset, lasting for one to three days; (4) small, transient corneal dendritic lesions of late-onset, lasting for one to three days; (4) vesicles, pustules, and crusts on the eyelids and their surroundings; and (5) pseudomembranous conjunctivitis in severe cases.

Given the pervasiveness of follicular conjunctivitis and preauricular adenopathy in all acute viral conjunctivitis and the ephemerality of dendritic lesions, primary infections are usually only identified when accompanied by vesicular lesions on the eyelid. Studies in developed countries have emphasized the progressive displacement of primary infections toward older ages with improvements in the population’s socioeconomic conditions^(5)^. Among adults, this condition manifests as an acute type of follicular conjunctivitis with vesicles and eyelid ulcers^(6,7)^. Under favorable conditions, viruses can reactivate and move peripherally through one of the three branches of the trigeminal nerve, promoting an endogenous infection of the integumentary system. When the eye is affected, viruses can take the following path: ophthalmic nerve, nasociliary nerve, long ciliary nerves, deep radial corneal nerves, and subepithelial nervous plexus. One interesting theory suggests that the colonized sensory ganglia would periodically release viruses into dermatomes, producing subclinical microfoci of infection that would be eliminated by defense mechanisms^(8)^. Only dermatomal changes that favor virus replication would promote the progression of these microfoci into lesions. Such changes, which may be associated with the release of inflammatory mediators like prostaglandins, would last for a limited time after a specific stimulus.

The clinical manifestations resulting from viral reactivation characterize the “recurrent ocular herpes,” and the agents allegedly triggering this event are fever, ultraviolet radiation, and eye trauma^(9)^. Its findings are divided into epithelial, stromal, and endothelial and its pathophysiology, categorized as infectious, immunological, or mixed.

### Epithelial Herpes

Dendritic keratitis, first described by Kipp (1880) among patients with Malaria and whose current name was later established by Hansen-Grut^(10)^, is considered the archetypal herpetic corneal infection. This condition initially presents as an epithelial plaque of opaque cells with dichotomized branches. In approximately 24 h, its center peels off, giving rise to a dendritic epithelial defect with terminal bulbs and edematous borders filled with viruses that can be stained exuberantly using the Rose Bengal dye^(11)^. Occasionally, the ulceration expands to the ameboid or geographic form, while some lesions exhibit a starry or punctate shape. Regardless of the shape, they can all be stained with fluorescein, given their de-epithelialized center. When left untreated, they disappear within one to three weeks.

The treatment of epithelial keratitis, the only purely infectious herpetic keratitis, includes the following alternative drugs, all of which administered for ten days: (1) acyclovir ophthalmic ointment (3%) five times daily; (2) acyclovir 400 mg orally five times daily; (3) valacyclovir 500 mg orally two times daily; and (4) famciclovir 250 mg orally two times daily. Acyclovir and valacyclovir have been the drugs of choice for the treatment of ocular herpes. Although both are very well tolerated, they are only useful for herpes simplex and zoster viruses. Valacyclovir is a prodrug that is immediately converted to acyclovir by intestinal and hepatic metabolism. Given that it requires less medication and does not contain lactose in its formulation, which causes intestinal discomfort in individuals intolerant to it, it is more convenient than acyclovir.

The average dosage of acyclovir in children is 30 mg/kg/day divided into three or more doses for ten days. Considering that this medication is available in Brazil only as tablets, children up to 2 years old receive half a tablet of acyclovir (200 mg) thrice daily; 2- to 4-yearold children receive one tablet of acyclovir (200 mg) thrice daily, and 4- to 12-year-old children receive half a tablet (500 mg) of valacyclovir twice daily. Tablets are crushed and mixed in juice or pasty substances, such as yogurt, to improve ingestion. Patients from 12 years old to adulthood receive acyclovir (400 mg) four times daily or the adult dose of valacyclovir. Although the Food and Drug Administration has not yet approved such drugs to pregnant women, they have been used widely without reports of harm to the fetus, at any stage of pregnancy^(12,13)^. Moreover, the amount of antiviral passed into the milk is equivalent to approximately 2% of the infant’s daily therapeutic dose. Therefore, there is no reason to assume that the mother’s treatment intoxicates the nursing baby^(14)^. We prefer the oral route for the antiviral agents because of their potential epithelial toxicity when applied topically.

When recurrences become frequent, the vicious cycle needs to be interrupted, considering that each episode facilitates the next^(3)^. For this purpose, acyclovir 400 mg orally twice daily or valacyclovir 500 mg orally daily is admi nistered for one year^(15-17)^, with children receiving half of the therapeutic dose. Among patients with a history of recurrent herpes, a prophylactic dose of acyclovir for appro ximately a year and a half has significantly decreased graft recurrence and transplant failure^(18)^.

Since acyclovir is eliminated primarily by the kidneys, usual dosages should only be modified in patients with severe renal impairment^(19)^. Despite the low toxicity of anti-herpetic agents, quarterly control of renal and liver function during long-term treatment is advisable.

### Stromal herpes

Clinical manifestations of stromal herpes result from the combination of viral replication and the host’s immune response to viral antigens at varying degrees. The following classification can help with the clinical diagnosis of and therapeutic planning for stromal herpes.

#### Subepithelial keratitis

**Figure 1:**
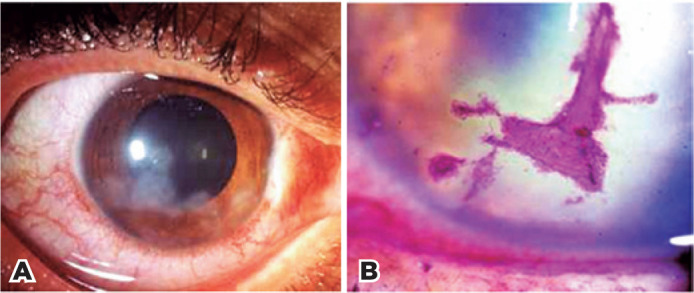
Subepithelial keratitis. A) Ghost scar. B) Ulcerative form (hot-iron mark).

As in any adenovirus keratitis, a white-gray subepithelial infiltrate may appear in the underlying superficial stroma a few days after the epithelial lesion´s appearance, which, in the case of herpes, assumes a nearly dendritic outline (Ghost reaction). These infiltrates indicate an immunological reaction to viral antigens retained at the corneal subepithelial nervous plexus (Figure 1A). They cause a foreign body sensation, sensitivity to light, and disappear spontaneously, leaving a scar that reveals the place of the epithelial involvement (Ghost scar)^(11)^. Although such infiltrates respond promptly to two drops of conventional corticosteroid daily, this treatment is only recommended when symptoms are intolerable since the total time of steroidal agent use is not predictable.

Depending on the intensity of inflammation, subepithelial keratitis can become ulcerated, with varying degrees of corneal thinning. The ulcer manifests as a stromal depression with an irregular edge resembling a skin mark made with a hot iron. The ulcer’s bed is vividly stained with Rose Bengal, but its epithelial edge retains the dye only in the presence of active infection (Figure 1B). The lesion can consume a considerable amount of stromal substance according to the intensity and number of phlogistic cycles, leading to a corneal perforation in extreme cases. Considering that such ulcers can coexist with epithelial viral infections, antivirals must be administered with corticosteroid therapy. Treatment starts with two to three daily instillations of topical corticosteroids, tapered with the inflammation improvement. Concomitantly, we provide therapeutic doses of acyclovir or valacyclovir for 15 days. After this period, we continue with a preventive dose of the antiviral agent until the steroid use cessation. A minimum treatment duration of 30 to 45 days is expectable. All clinical manifestations of this morbidity usually cause a decrease in corneal sensitivity, probably due to inflammatory or scarring injury to the subepithelial nervous plexus. The distribution and intensity of hypoesthesia depend on the location and severity of nerve damage. Vision reduces with scar density and corneal surface irregularity.

Differential diagnoses include geographic and neurotrophic keratopathies. The ulcerated form of subepithelial keratitis has intense Rose Bengal staining, weak fluorescein staining, and variable stromal inflammatory thinning; geographic ulcers have the opposite dyeing pattern with no stromal involvement. Thus, the only common element between these two entities is the irregular border. Neurotrophic Keratopathy is discussed in the next section.

#### Neurotrophic keratopathy

Neurotrophic keratitis is an indolent epithelial defect due to a deficiency in corneal innervation. It does not respond to antiviral and anti-inflammatory agents. The edge of the lesion, consisting of stacked epithelial cells with rolled configuration, limits the bare stroma that stains vividly with the Bengal rose. Considering that the herpetic epithelial lesion is a source of permanent innervation damage, it is plausible that, in some cases, it may progress to neurotrophic keratitis. However, the relative importance of this morbidity in the herpes simplex clinical picture’s characterization is debatable. Many of the diagnoses of neurotrophic keratitis are mere manifestations of drug toxicity to topical antivirals, where the elimination of the toxic agent and use of therapeutic contact lenses solve the problem. In the past, these toxic reactions were also confused with the obscure entity called a meta-herpetic ulcer. Its principal differential diagnosis is ulcerated subepithelial keratitis, which exhibits the same exuberant stromal staining with Rose Bengal.

#### Disciform keratitis

**Figure 2:**
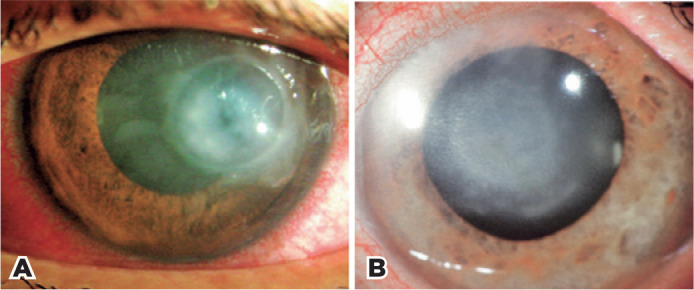
Disciform keratitis. A) Active form. B) Scar with multiple rings.

Under this name, Fuchs retitled (1901) a peculiar form of keratitis known as Arlt’s *abscessus siccus*^(20,21)^, characterized by a gray disk-shaped opacity of the middle layers of the cornea, in the center of which lies a small, deeply clouded speck. The periphery of the disk is sharply delimited by a darker grayish circular line (Figure 2A), which, in many cases, exhibits concentric rings^(22)^. Busacca^(23)^, confirming the previous findings of Wagner^(20)^, showed that this condition’s essential anatomopathological trait is hyaline or granular necrosis of a circumscribed group of corneal lamellae, followed by an inflammatory reaction from the neighboring tissue.

The dark gray ring corresponds to Morawiecki’s immune ring (inappropriately called Wessely’s ring), an annular intrastromal deposit of antigen-antibody complexes to which lymphocytes and plasmatic cells adhere^(24,25)^. Disciform keratitis itself is amazingly analogous to the immunological super-rings described by Breebaart and James-Witte, which represent a variety of anaphylactic interstitial keratitis^(26)^. As such, Fuchs’ disciform keratitis is a type of inflammatory morbidity strongly linked to an immunological reaction affecting the corneal stroma. Given that Fuchs had no such information during his time, he believed that an exogenous bacterial infection caused the keratitis, the central opaque speck being the microorganisms’ entry point. This misconception might have been the origin of the dogmatic statement that infection always travels from the surface to the deep stroma^(21)^. Considering the current understanding that herpetic corneal infections are mostly of endogenous origin and that viruses can reach the epithelial layer and the trabecular meshwork via innervation^(27)^, we wonder why they would not be able to find their way directly into the deep stroma.

Disciform keratitis leads to Descemet’s membrane folds, keratic precipitates, iridocyclitis, and, occasionally, ocular hypertension. The epithelium may be normal or exhibit bullous edema. When left untreated, it spontaneously regresses within two to six months. In the course of the disease, superficial or deep blood vessels can invade the lesion, leaving as a sequel variable degree of vascularization and stromal opacity (Figure 2B). The typical symptoms are light sensitivity and reduced vision. Disciform keratitis is not exclusive to HSV and can occur in herpes zoster, chickenpox, vaccinia, measles, and toxicity. To confirm herpetic etiology, the presence of a scar, history, or laboratory test suggesting herpes simplex is needed.

The etiopathogenesis and nomenclature of this keratitis have remained a matter of dispute since the nineteenth century. Some believe that this condition is a toxic^(28)^ or immunological^(29)^ stromal reaction to HSV particles, whereas others maintain that it is a consequence of the endothelium’s direct viral infection^(30)^. The latter group classifies this condition as herpetic endotheliitis, with the disc indicating edema secondary to endothelial distress. However, the small prevalence of positive viral cultures retrieved from the aqueous, the relatively small destruction of endothelial cells, the favorable response to corticotherapy, the ineffectiveness of antiviral therapy, the pathological findings, and the sequels do not support the endothelium’s direct-viral-damage conjecture^(23,28)^. This controversy might stem from the exclusion of immunological rings from the definition of disciform keratitis. Grayson^(31)^ defines the condition as an oval or circular stromal edema, with some cellular infiltration, under an intact epithelium, or a dendritic lesion. Since the definition does not allude to the gray ring, it opens a window for confusion between the disk-like edema of certain endotheliitis and disciform keratitis.

Treatment consists of corticosteroid drops at usual concentrations. Without consensus regarding the daily frequency of eye drops use, the prevailing philosophy is that steroids should be avoided whenever symptoms are well-tolerated. However, when indicated, steroids should be used at the lowest possible dosage to control inflammation. We can start with four drops daily. As soon as the condition improves, we change it to three, two, and then one, continuing with the smallest frequency for about three months. Early withdrawal of the anti-inflammatory agent can cause the inflammation to rebound with tissue necrosis. A small proportion of patients require longer treatment times, with corticosteroid solutions diluted 8 to 16 times.

#### Interstitial keratitis

Interstitial keratitis also appears in the literature as parenchymal keratitis^(22)^, immunological keratitis^(30)^, and non-necrotic stromal keratitis^(32)^. Individuals with such a condition usually have a history of several episodes of dendritic or disciform keratitis^(31)^. Its primary differential diagnoses are interstitial keratitis of syphilis, tuberculosis, leprosy, and Cogan’s syndrome.

Interstitial keratitis corresponds to a dense grayish-white infiltrate of mononuclear cells embedded into the corneal interlamellar spaces, accompanied by deep fascicular vascularization with varying degrees of stromal thinning. At the height of inflammation, the cornea becomes so opaque that recognition of the iris grows impossible. The vision becomes of hand movements. Recovery begins at the corneal periphery, with the progressive restoration of transparency toward the center. Eventually, the transparency recovers with a nebula and a residual web of atrophied vessels as sequelae^(22)^. Wessely (1911) reproduced this event experimentally approximately 10 to 14 days after injecting equine serum into the rabbit’s corneas. This particular form of immunological reaction is known as Wessely’s Phenomenon^(25,26)^.

**Figure 3:**
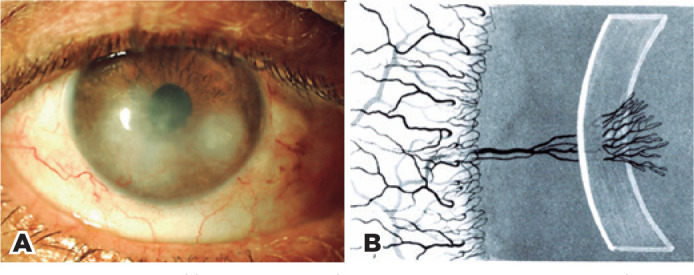
Interstitial keratitis. A) Relapsing stage. B) Deep fascicular vascularization

In clinical practice, patients often present with the recurrent form of interstitial keratitis, with the cornea showing deep engorged stromal vessels in a brush-like pattern surrounded by a diffuse infiltrate and mild stromal edema (Figure 3). They often complain of a foreign body sensation in the eye, tearing, and sensitivity to light. Treatment for interstitial keratitis is like that for disciform keratitis.

Fonte: Busacca A. Kératite disciforme. Biomicroscopie et Histologie de

L’oeil. Zurich: Schweizer Druck und Verlagshaus SA; 1952.^(23)^

#### Necrotizing stromal keratitis

**Figure 4:**
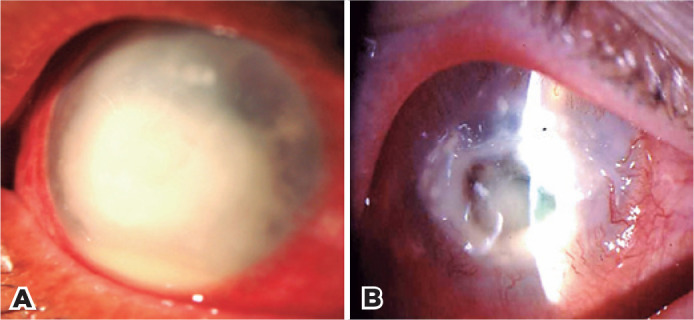
Necrotizing keratitis. A) Non-ulcerative stage. B) Ulcerative stage.

Necrotizing keratitis denotes a creamy white necrotic mass of variable thickness inside the corneal stroma, accompanied by corneal thinning, vascularization, and, occasionally, ocular perforation (Figures 4A and 4B). Anterior uveitis is almost constant and may exhibit retrocorneal membranes, hypopyon, synechiae, cataracts, and glaucoma. This disease has a natural course of 2 to 12 months. The predominant symptoms are severe pain and low vision. Necrotizing keratitis seems to be caused by an extreme immune response to viral material that has penetrated the deep stroma^(33)^. Viruses are rarely isolated from diseased corneas, except when epithelial lesions were treated with large amounts of corticosteroids without antiviral coverage^(33)^. In experimental rabbit models, the virus needed to multiply for a week before the stromal disease was evident^(34)^. Antiviral administration to the eye during the first two days of infection prevented stromal disease. However, they were useless when provided after stroma colonization. On the other hand, corticosteroid became useful and riskless of worsening the disease in this scenario. The host’s response to the virus and its antigens produced severe immunological stromal keratitis^(35)^. Evidence in both rabbits or humans has suggested that the herpes virus can insert glycoproteins into the host membrane, making it antigenically unfamiliar and, therefore, creating the conditions necessary for a chronic autoimmune reaction^(36,37)^.

As the degree of influence of viral replication in triggering the immune response is still unknown, there is no consensus on this ailment’s best treatment. A common approach among Americans has been a combination of topical corticosteroids and topical trifluridine (1%), drop for drop until the anti-inflammatory agent’s frequency reduces to less than four times daily. From that time onwards, the antiviral agent is decreased at a faster pace or discontinued. When the steroid application frequency reaches its minimum, diluted dosages of a commercially available product have occasionally been used^(11)^. Even though controlled studies have endorsed this approach^(38,39)^, the optimal dose and duration of therapy for both steroidal and antiviral agents are unknown.

Our treatment strategy starts with four drops of corticosteroid daily, which are gradually decreased in frequency as inflammation subsides. Simultaneously, we use therapeutic doses of acyclovir or valacyclovir for 15 days. The antiviral treatment aims to eradicate any virus that might be replicating into the stroma in the hope of attenuating additional immune response. As we do not have topical trifluridine in Brazil, after the expiration of conventional treatment, we maintain the oral antiviral, in a preventive dose, until the abolition of the corticotherapy. The minimum treatment time is about three months. Preventive therapy stems from our reluctance to confront the dogma that we should not use corticosteroids alone when there is a risk of viral reactivation^(40)^. We acknowledge, though, that such policy is questionable. After the antiviral treatment, the infection is gone, and the steroidal agents are being used in quantities that seem to be too small to elicit virus replication^(9,41)^. As laboratory tests are not very accessible, herpetic keratitis diagnosis continues to rely upon clinical findings.

### Endothelial Herpes (Herpetic Endotheliitis)

Endotheliitis is an inflammation of the corneal endothelium manifested by keratic precipitates and stromal edema. It can be classified as primary when inflammation starts in the endothelium and secondary when it spills over from neighboring structures, such as the cornea and anterior chamber. The main symptoms are sensitivity to light, mild pain, and reduced vision. Endotheliitis can affect specific regions of the endothelium, causing focal stromal edema, or involve the entire endothelial layer, causing abrupt and generalized bullous keratopathy. Some endotheliites are expansive, moving from the limbus to the corneal center, led by a line of keratic precipitates^(42-44)^. In cases involving corneal transplantation, the keratic precipitates colonize both the donor and recipient endothelium^(44)^, contrasting with the real rejection where the precipitates are confined to the graft^(45)^.

The primary form of endotheliitis is found in various viral infections (herpes simplex, varicella-zoster, and cytomegalovirus) and toxic phenomena^(46)^. Given that this type of endoteliitis tends to respond satisfactorily to corticotherapy, it is reasonable to suppose that it originates from an immunological reaction to the invading agent’s substance. On the other hand, there are cases refractory to steroids that respond only to antiviral therapy. Those cases generally manifest as an expansive endoteliitis in which HSV particles can be retrieved from the anterior chamber^(42-44)^. In such cases, the disease probably results from direct viral damage.

Endotheliitis is usually diagnosed based on history, the presence of scarring lesions suggestive of herpes simplex, and, ideally, on a positive polymerase chain reaction test of the aqueous humor^(47)^. Treatment consists of corticosteroid drops for 30 to 45 days, starting with four drops daily or six, in those with severe and generalized edema. The frequency of instillation decreases with the inflammation’s decaying. In cases refractory to corticotherapy or those with a strong suspicion of active herpetic infection, such as an expansive endotheliitis, the usual dose of antivirals is provided for ten days, and followed by a prophylactic dose until the withdrawal of the steroid agent. In the absence of clinical improvement, an analysis of aqueous humor helps explore diagnostic alternatives.
